# Arterial Stiffness and Early Vascular Damage in Ankylosing Spondylitis Regardless of Cardiovascular Risk Factors: A Cross-Sectional Study

**DOI:** 10.31138/mjr.030226.rea

**Published:** 2026-06-16

**Authors:** Miroslav Markov, Maria Dimova, Tsvetoslav Georgiev

**Affiliations:** Medical University Varna Prof Dr Paraskev Stoyanov - First Department of Internal Medicine, Varna, Bulgaria

**Keywords:** ankylosing spondylitis, arterial stiffness, pulse wave analysis, carotid intima-media thickness, cardiovascular diseases

## Abstract

**Background:**

Our aim is to evaluate subclinical vascular damage in patients with ankylosing spondylitis using one-point echo-tracking-derived arterial stiffness indices and carotid intima–media thickness (CIMT), and to explore associations with inflammatory activity and traditional cardiovascular risk scores.

**Methods:**

This cross-sectional study included 83 patients with AS and 31 age- and sex-matched controls without inflammatory or cardiovascular disease. All participants underwent high-resolution ultrasound of the common carotid artery with one-point echo-tracking assessment of arterial stiffness parameters, including β-stiffness index, local pulse wave velocity (PWVβ), Peterson’s elastic modulus (Ep), arterial compliance, and augmentation index, alongside CIMT measurement. Ten-year cardiovascular risk was estimated using the Framingham Risk Score and SCORE2.

**Results:**

Traditional cardiovascular risk estimates were comparable between groups (Framingham risk: 3.7 [2.0–7.5]% vs 5.25 [2.5–8.0]%, p = 0.378; SCORE2: 6.7 [3.5–10.5]% vs 7.3 [3.0–11.0]%, p = 0.718). In contrast, patients with AS demonstrated significantly increased arterial stiffness, with higher β-stiffness index (7.0 [5.4–8.6] vs 6.05 [5.2–6.9], p < 0.001), PWVβ (5.8 [5.2–6.5] vs 5.45 [4.9–5.9] m/s, p < 0.001), and Ep (90 [68–112] vs 78.5 [66–90] kPa, p < 0.001). CIMT did not differ significantly between groups. Body mass index independently predicted arterial stiffness (PWVβ standardized β = 0.41, p < 0.001).

**Conclusions:**

Ankylosing spondylitis is characterised by early functional vascular impairment despite preserved traditional cardiovascular risk profiles. One-point echo-tracking–derived arterial stiffness indices identify subclinical vascular damage beyond conventional risk assessment and may improve cardiovascular risk stratification in AS.

## BACKGROUND

Ankylosing spondylitis (AS) is associated with a substantially increased cardiovascular risk that exceeds the burden expected from traditional risk factors alone.^1^ Chronic systemic inflammation plays a pivotal role in this process by inducing endothelial dysfunction, oxidative stress and adverse vascular remodelling, thereby accelerating atherosclerosis and premature arterial ageing.^2,3^ These inflammation-driven vascular changes create a substrate for subclinical cardiovascular damage long before the onset of overt clinical events.^4,5^

Large population-based and cohort data consistently demonstrate higher rates of ischemic heart disease, cerebrovascular events and cardiovascular mortality in patients with AS compared with the general population, confirming the clinical relevance of inflammation-mediated vascular injury.^6–8^ Importantly, conventional cardiovascular risk prediction models frequently underestimate this excess risk, highlighting the need for complementary approaches capable of detecting early, subclinical vascular alterations.

Chronic inflammation drives structural remodelling of the arterial wall, leading to increased arterial stiffness through elastin degradation, vascular smooth muscle cell proliferation and alterations of the extracellular matrix.^2,9,10^ Elastin fragmentation within the medial layer shifts haemodynamic load towards collagen fibres, which have markedly lower compliance, thereby reducing arterial elasticity.

The resulting loss of arterial elasticity alters pulse wave propagation, increases systolic and pulse pressure and augments left ventricular afterload, thereby linking arterial stiffness to adverse cardiovascular outcomes.^11–13^ Although these processes accompany ageing, they are accelerated by cardiovascular risk factors and chronic systemic inflammation.^2,12,14^

Assessment of arterial stiffness is based on biomechanical indices reflecting the elastic properties of the arterial wall, derived from ultrasound-based measurements of arterial diameter throughout the cardiac cycle combined with blood pressure values.^15^ One-point echo-tracking–derived arterial stiffness indices represent validated local biomechanical parameters that demonstrate good correlation with carotid–femoral pulse wave velocity and offer precise assessment of arterial wall properties. Parameters that are used are the stiffness index β, Peterson’s elastic modulus (Ep), arterial compliance (ACo) and augmentation index (AI), which collectively describe arterial elasticity, pressure– diameter relationships and pulse wave reflection.^16,17^ Local pulse wave velocity (PWVβ) can be calculated without external distance measurement, allowing functional assessment of arterial stiffness at a single vascular site.^17^

Although carotid–femoral PWV is considered the reference method for global arterial stiffness assessment, its reliance on external distance measurement and inclusion of heterogeneous vascular segments may limit accuracy in certain clinical settings.^13–16^ These limitations have driven increasing interest in one-point assessment using echo-tracking technology, which enables precise and reproducible local evaluation of arterial stiffness while avoiding distance-related errors.^17^

In addition to functional indices, ultrasound measurement of carotid intima–media thickness (CIMT) provides complementary structural information on early atherosclerotic changes.^18^ Combined assessment of arterial stiffness and CIMT allows integrated evaluation of functional and structural vascular damage, particularly relevant in inflammatory diseases characterised by accelerated vascular ageing. Echo-tracking, based on high-frequency B-mode ultrasound, facilitates simultaneous assessment of local arterial stiffness and CIMT with high reproducibility and clinical applicability.^19^

Despite the well-documented association between ankylosing spondylitis and excess cardiovascular risk, early vascular involvement frequently remains subclinical and may not be adequately identified by conventional cardiovascular risk models. Evaluation of arterial stiffness, together with carotid intima-media thickness, offers the opportunity to detect functional and structural vascular alterations at an early stage, particularly in the setting of chronic inflammation. Accordingly, the present study aimed to assess the degree of subclinical vascular damage in patients with ankylosing spondylitis compared with control subjects without inflammatory rheumatic disease, using one-point echo-tracking-derived arterial stiffness indices and carotid intima–media thickness measurement. In addition, associations with inflammatory activity and traditional cardiovascular risk scores were explored.

## METHODS

### Study population

This cross-sectional study included 114 participants: 83 patients with ankylosing spondylitis (AS) and 31 age- and sex-matched controls. AS was diagnosed according to the Assessment of SpondyloArthritis International Society (ASAS) classification criteria for axial spondyloarthritis. Control subjects were recruited from individuals undergoing routine health evaluations and hospital staff volunteers. Inclusion criteria required absence of inflammatory rheumatic disease and absence of established cardiovascular disease, including ischemic heart disease, heart failure, cerebrovascular disease, or peripheral arterial disease. The presence of cardiovascular risk factors (e.g., arterial hypertension, dyslipidaemia, or smoking) was not considered an exclusion criterion, in order to ensure comparability with the AS cohort and reflect a non-inflammatory general population sample.

Participants were recruited from the Departments of Rheumatology and Internal Medicine, St. Marina University Hospital, Varna, Bulgaria, between 2023 and 2024. The study was approved by the Ethics Committee of Medical University of Varna (Protocol 133/22.06.2023), and written informed consent was obtained from all participants.

Exclusion criteria included documented ischemic heart disease, cerebrovascular or peripheral arterial disease, heart failure, significant arrhythmias, diabetes mellitus, chronic kidney disease (stage ≥3), active infection, malignancy, pregnancy, or inability to undergo ultrasonographic examination.

### Clinical and inflammatory assessment

Demographic characteristics, smoking status, disease duration and current therapy were recorded. Blood pressure was measured under standardised conditions.

Inflammatory activity in AS patients was assessed using the Ankylosing Spondylitis Disease Activity Score with C-reactive protein (ASDAS-CRP),^20^ calculated according to the official ASAS formula. C-reactive protein (CRP) and erythrocyte sedimentation rate (ESR) were measured using standard laboratory methods. In addition to ASDAS-CRP, disease activity was assessed using the Bath Ankylosing Spondylitis Disease Activity Index (BASDAI).^21^

### Ultrasonographic assessment

All participants underwent ultrasound examination of the right common carotid artery using a high-resolution ultrasound system (Aloka Hitachi Prosound α7) with a linear high-frequency transducer. Measurements were performed approximately 2 cm proximal to the carotid bifurcation in a longitudinal plane.^22^

Carotid intima–media thickness (CIMT) was measured at the posterior wall of the common carotid artery in plaque-free segments. Three measurements obtained during different cardiac cycles were averaged for analysis.

**Figure 1.**
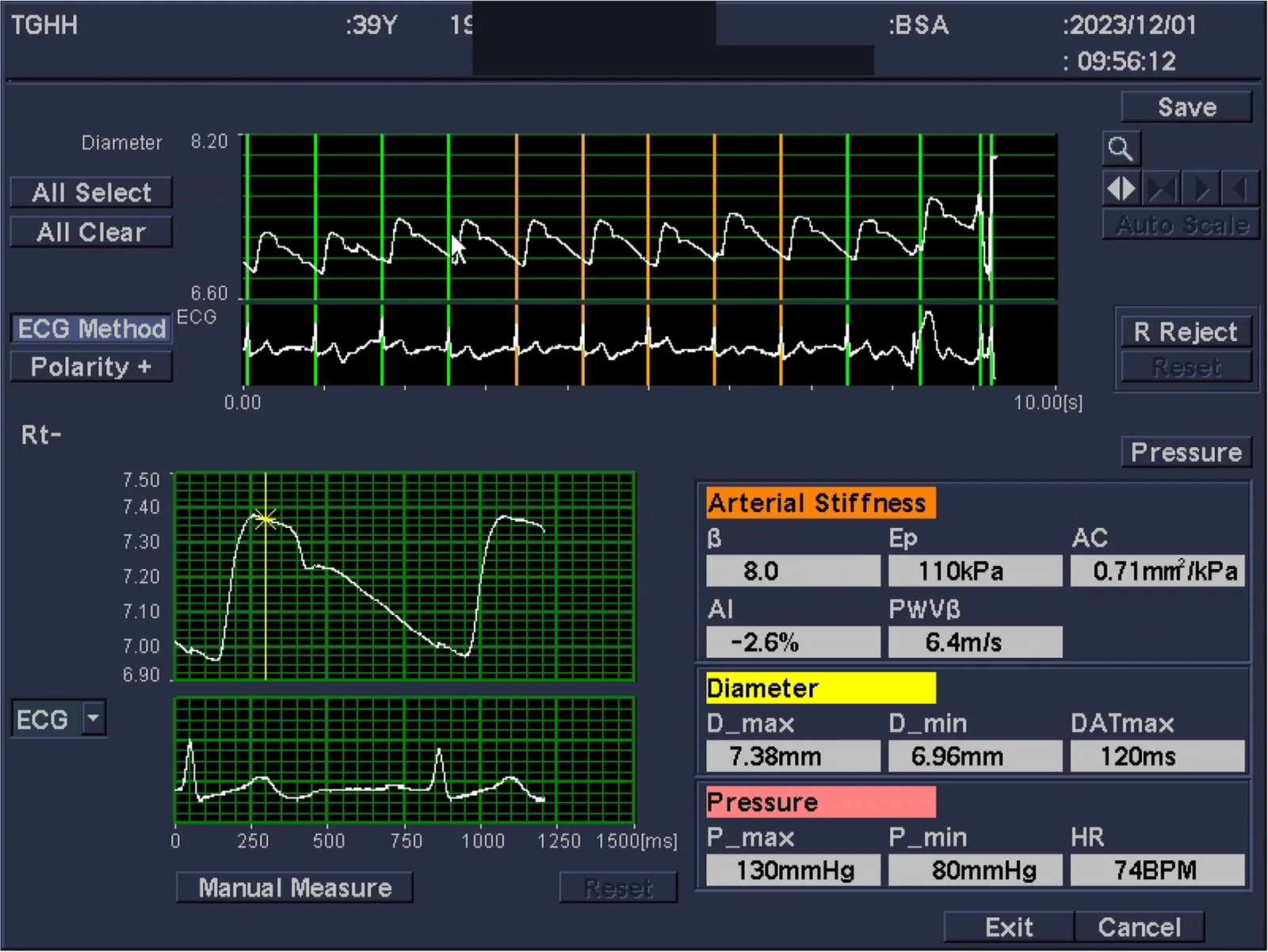
One-point echo-tracking assessment of arterial stiffness at the level of the common carotid artery. PWV: pulse wave velocity; Β: stiffness index beta; AI: augmentation index; Ep: pressure-strain elastic modulus (Peterson’s modulus); AC: arterial compliance; Dmin: minimal arterial diameter; Dmax: maximal arterial diameter; Pmax: maximal arterial pressure; Pmin: minimal arterial pressure; HR: heart rate.

Arterial stiffness was assessed using one-point echo-tracking technology, with arterial diameter changes recorded synchronised with electrocardiographic tracing.^23^ The following biomechanical indices were automatically calculated by the integrated software using simultaneously entered blood pressure values (**Figure 1**).

The formulas and physiological interpretation of echo-tracking–derived arterial stiffness indices are presented in **Table 1**.

**Table 1.** Biophysical indices of arterial stiffness: formulas and physiological interpretation.

**Parameter**	**Formula**	**Physiological meaning**
**Stiffness index (β)**	β = ln(Ps / Pd) / [(Ds -Dd) / Dd]	Reflects arterial wall deformation relative to logarithmic changes in blood pressure; marker of intrinsic arterial stiffness.
**Pressure-strain elastic modulus (Ep)**	Ep = (Ps - Pd) / [(Ds- Dd) / Dd]	Peterson’s elastic modulus; estimates arterial wall resistance to pressure-induced deformation.
**Arterial compliance (ACo)**	ACo = ρ (Ds^2^ -Dd^2^) / [4 × (Ps - Pd)]	Describes the ability of the artery to expand in response to pressure changes; inversely related to arterial stiffness and PWV.
**Augmentation index (AIx)**	AIx = ΔP / PP × 100	Percentage of pulse pressure attributable to reflected waves; indirect marker of arterial stiffness and wave reflection.
**Local pulse wave velocity (PWVβ)**	PWVβ = √[(β × Pmin) / (2ρ)]	Local estimation of pulse wave velocity derived from β-index; does not require external distance measurement.

Ps: systolic blood pressure; Pd: diastolic blood pressure; Ds- arterial diameter during systole; Dd: arterial diameter during diastole; ΔP: augmentation pressure; PP: pulse pressure; Pmin: minimal arterial pressure; ρ: blood density (assumed 1050 kg/m^3^); ln: natural logarithm.

### Cardiovascular risk assessment

Ten-year cardiovascular risk was estimated using validated risk prediction models. The Framingham Risk Score and SCORE2 were calculated for each participant according to current European Society of Cardiology recommendations.^24–26^ For participants aged ≥70 years, SCORE2-OP was applied.^27^

### Statistical analysis

Statistical analysis was performed using IBM SPSS Statistics version 27.0. Normality of distribution was assessed using the Shapiro–Wilk test and additionally confirmed using the Kolmogorov–Smirnov test for the AS group (n = 83). As the majority of continuous variables were not normally distributed, they are presented as median (interquartile range). Continuous variables with normal distribution (when applicable) are presented as mean ± standard deviation (SD). Between-group comparisons (AS vs controls) were performed using the Mann-Whitney U test for continuous variables and Pearson’s chi-square test for categorical variables. Associations between arterial stiffness indices, CIMT, inflammatory markers and cardiovascular risk scores were analysed using Spearman’s correlation. Multivariable linear regression was applied to identify independent predictors of arterial stiffness parameters and cardiovascular risk scores. Covariates included in the multivariable models were selected based on established determinants of arterial stiffness reported in the literature (age, sex, body mass index, smoking status, and hypertension) and on their clinical relevance, while considering the sample size to avoid model overfitting. A p-value <0.05 was considered statistically significant.

## RESULTS

### Baseline characteristics and inflammatory burden

Patients with ankylosing spondylitis (AS) and control subjects were well matched with respect to demographic characteristics and major cardiovascular risk factors. Mean age was comparable between the AS group (48.08 ± 10.66 years) and controls (51.5 ± 10.5 years; p = 0.508), as was body mass index (27.99 ± 5.54 vs 27.31 ± 4.80 kg/m^2^, p = 0.319). The proportion of male participants did not differ significantly between groups (66.3% in AS vs 54.8% in controls; p = 0.260). Similarly, no significant differences were observed in the prevalence of arterial hypertension (27.7% vs 35.5%; p = 0.446) or current smoking status (55.4% vs 64.5%; p = 0.854).

Against this background of comparable baseline cardiovascular risk, patients with ankylosing spondylitis exhibited a substantially higher inflammatory burden. Median erythrocyte sedimentation rate was significantly higher in the AS group (32 [18–41] mm/h), accompanied by elevated C-reactive protein levels (4.0 [1.5–9.0] mg/L), compared with controls (21.5 [12–30] mm/h and 2.11 [1.0–4.0] mg/L, respectively) (ESR: p = 0.004; CRP: p = 0.026). Median disease duration was 10 years, and median ASDAS-CRP and BASDAI values indicated overall moderate disease activity. Within the ankylosing spondylitis cohort, 60.2% of patients were receiving anti-TNF therapy, 14.5% IL-17 inhibitors, and 7.2% JAK inhibitors, while 18.1% were biologic-naïve. All treatment regimens were stable at the time of vascular assessment.

### Lipid profile and global cardiovascular risk scores

Despite this pronounced inflammatory activity, no significant differences were observed between groups with respect to lipid parameters, including total cholesterol, triglycerides, LDL-cholesterol and HDL-cholesterol (all p > 0.05), with comparable mean values across groups. In parallel, global cardiovascular risk estimates did not differ significantly, as assessed by the Framingham risk score (3.7 [2.0–7.5]% vs 5.25 [2.5–8.0]%, p = 0.378) and SCORE2 (6.7 [3.5–10.5]% vs 7.3 [3.0–11.0]%, p = 0.718) (**Table 2**). No significant direct association was observed between inflammatory markers and arterial stiffness indices.

**Table 2.** Clinical, laboratory and cardiovascular characteristics of patients with ankylosing spondylitis and control subjects.

**Parameter**	**AS (n=83)**	**Controls (n=31)**	**p value**
**Age (years)**	48.08 ± 10.66	51.5 ± 10.5	0.508
**Male sex, n (%)**	55 (66.3%)	17 (54.8%)	0.260
**BMI (kg/m^2^)**	27.99 ± 5.54	27.31 ± 4.80	0.319
**Arterial hypertension, n (%)**	23 (27.7%)	11 (35.5%)	0.446
**Current smokers, n (%)**	46 (55.4%)	20 (64.5%)	0.854
**ESR (mm/h)**	32 (18–41)	21.5 (12–30)	0.004
**CRP (mg/L)**	4.0 (1.5–9.0)	2.11 (1.0–4.0)	0.026
**Total cholesterol (mmol/L)**	5.10 (4.50–5.80)	4.97 (4.30–5.60)	0.582
**Triglycerides (mmol/L)**	1.17 (0.90–1.60)	1.23 (0.90–1.70)	0.392
**LDL-cholesterol (mmol/L)**	3.17 ± 0.96	2.78 ± 0.85	0.093
**HDL-cholesterol (mmol/L)**	1.43 ± 0.36	1.51 ± 0.40	0.151
**Framingham risk score (%)**	3.7 (2.0–7.5)	5.25 (2.5–8.0)	0.378
**SCORE2 (%)**	6.7 (3.5–10.5)	7.3 (3.0–11.0)	0.718

Data are presented as mean ± SD or median (interquartile range).

### Vascular ultrasound findings

Ultrasound assessment revealed marked alterations in arterial stiffness parameters in patients with ankylosing spondylitis compared with control subjects. Specifically, the AS group demonstrated significantly higher β-stiffness (7.0 [5.4–8.6] vs 6.05 [5.2–6.9], p < 0.001), pulse wave velocity (5.8 [5.2–6.5] vs 5.45 [4.9–5.9] m/s, p < 0.001), and elastic modulus Ep (90 [68–112] vs 78.5 [66–90] kPa, p < 0.001). In contrast, carotid intima-media thickness was numerically higher in patients with AS but did not differ significantly from controls (0.54 [0.46–0.69] vs 0.51 [0.43–0.58] mm p= 0.062), while arterial compliance also did not differ significantly between groups (p= 0.181) (**Table 3**).

**Table 3.** Ultrasound-derived arterial stiffness and carotid parameters in patients with ankylosing spondylitis and control subjects.

**Parameter**	**AS (n=83)**	**Controls (n=31)**	**p value**
**CIMT (mm)**	0.54 (0.46–0.69)	0.51 (0.43–0.58)	0.062
**β-stiffness index**	7.0 (5.4–8.6)	6.05 (5.2–6.9)	<0.001
**Pulse wave velocity (m/s)**	5.8 (5.2–6.5)	5.45 (4.9–5.9)	<0.001
**Arterial compliance (mm^2^/kPa )**	0.86 (0.70–1.03)	0.92 (0.62–1.22)	0.181
**Elastic modulus Ep (kPa)**	90 (68–112)	78.5 (66–90)	<0.001

Data are presented as median (interquartile range).

### Correlation analyses and multivariable predictors of arterial stiffness

To further elucidate the relationship between inflammation, disease activity and vascular alterations, correlation analyses were performed within the ankylosing spondylitis group. Markers of inflammation were closely linked to disease activity, with ASDAS-CRP showing significant positive correlations with both ESR (ρ = 0.579, p < 0.001) and CRP (ρ = 0.740, p < 0.001). In contrast, BASDAI did not show significant correlations with inflammatory markers, ultrasound-derived arterial stiffness parameters or CIMT (all p > 0.05), indicating that patient-reported disease activity was not directly associated with vascular involvement.

In contrast, isolated inflammatory markers did not demonstrate direct linear associations with ultrasound-derived arterial stiffness indices. Notably, disease activity assessed by ASDAS-CRP was inversely correlated with arterial compliance (ρ = −0.275, p = 0.037), although the association did not reach statistical significance after adjustment for age, sex, smoking status, and disease duration, indicating a modest independent effect (B = −0.040, standardised β = −0.222, p = 0.110).

Consistent with this observation, ultrasound-derived stiffness parameters exhibited strong internal coherence. β-stiffness, pulse wave velocity and elastic modulus were very strongly intercorrelated (all p < 0.001), whereas arterial compliance showed significant inverse associations with these indices. Carotid intima– media thickness demonstrated moderate correlations with functional stiffness measures, including β-stiffness (ρ = 0.523), PWV (ρ = 0.545) and Ep (ρ = 0.533), all p < 0.001. In contrast, traditional cardiovascular risk scores were primarily driven by age and showed no meaningful associations with arterial stiffness parameters, underscoring the ability of vascular ultrasound to capture subclinical vascular dysfunction in addition to traditional cardiovascular risk assessment.

To identify independent determinants of ultrasound-derived vascular stiffness, multivariable linear regression analyses were performed in patients with ankylosing spondylitis. Body mass index emerged as a consistent independent predictor of arterial stiffness across multiple parameters, including PWVβ, β-stiffness, and Ep (**Table 4**).

**Table 4.** Multivariable linear regression analyses of arterial stiffness parameters in patients with ankylosing spondylitis.

**Variable**	**PWV β**	**p**	**β-stiffness β**	**P**	**Ep β**	**p**
**Age**	−0.09	0.370	−0.23	**0.021**	−0.14	0.167
**Sex**	0.15	0.156	0.15	0.130	0.10	0.327
**Smoking**	−0.05	0.607	−0.04	0.715	−0.09	0.397
**Hypertension**	0.00	0.992	0.08	0.422	0.09	0.394
**BMI**	**0.41**	**<0.001**	**0.38**	**<0.001**	**0.42**	**<0.001**

## DISCUSSION

The present study demonstrates that patients with ankylosing spondylitis exhibit pronounced functional vascular abnormalities consistent with increased arterial stiffness, despite comparable traditional cardiovascular risk profiles and largely preserved structural markers of atherosclerosis. Using one-point echo-tracking technology, we show that β-stiffness, local pulse wave velocity and Peterson’s elastic modulus are significantly elevated in AS compared with matched controls, while carotid intima–media thickness remains only borderline increased. These findings provide evidence that functional vascular impairment may precede overt structural atherosclerotic changes in ankylosing spondylitis and may remain undetected by conventional cardiovascular risk assessment tools. Similar patterns have been reported in several AS cohorts, in which increased PWV, β-stiffness or wave reflection indices were observed despite limited differences in classical risk factors or CIMT.^28–30^ Complementary evidence from cardiovascular magnetic resonance further supports the presence of increased aortic stiffness in AS, suggesting that vascular involvement is not confined to a single arterial territory.^31^

### Functional vascular ageing despite comparable traditional risk profiles

A key observation of the present study is the dissociation between systemic inflammation and traditional lipid-based cardiovascular risk estimates. Despite significantly higher ESR and CRP levels in AS, lipid parameters, Framingham risk score and SCORE2 did not differ between patients and controls. This finding is consistent with previous reports indicating that conventional risk algorithms may underestimate cardiovascular risk in inflammatory rheumatic diseases,^32^ as they do not account for inflammation-driven vascular remodelling and accelerated arterial ageing. In this context, the marked increase in ultrasound-derived stiffness indices observed in our AS cohort underscores the added value of vascular imaging in detecting early cardiovascular damage in the context of traditional risk estimation, a concept also supported by prior ultrasound- and tonometry-based studies in AS.^29,30^

Importantly, carotid intima–media thickness did not differ significantly between groups, suggesting that structural atherosclerotic burden was still limited in this relatively middle-aged cohort. This observation aligns with studies reporting preserved or only modestly increased CIMT in AS, despite evidence of functional vascular impairment.^33,34^ Together, these findings support the concept that arterial stiffness reflects an earlier stage of vascular involvement, capturing functional alterations in the arterial wall that may precede measurable intimal thickening. The moderate correlations observed between CIMT and stiffness parameters further indicate that functional and structural vascular changes are related but not interchangeable, representing different stages along the continuum of vascular ageing.

### Inflammation, disease activity, and vascular dysfunction

Chronic inflammation is a central driver of vascular remodelling in ankylosing spondylitis, acting through multiple mechanisms including endothelial dysfunction, extracellular matrix degradation and altered smooth muscle cell phenotype. However, in the present study, isolated inflammatory markers (CRP and ESR) did not show direct linear associations with arterial stiffness indices. This observation is in line with several reports showing weak or absent associations between circulating inflammatory markers and PWV in AS, ^33,35^ and supports the notion that vascular damage may reflect cumulative inflammatory exposure rather than transient inflammatory activity captured at a single time point. The dissociation between systemic inflammation and conventional cardiovascular risk estimates may be partly explained by osteoimmunological mechanisms, whereby chronic cytokine-driven immune activation induces long-term vascular remodelling and arterial stiffening through pathways analogous to inflammation-mediated bone remodelling, independent of classical atherosclerotic risk factors.^36^

Disease activity assessed by ASDAS-CRP showed a modest inverse association with arterial compliance. In contrast, BASDAI showed no association with inflammatory markers or vascular parameters, reinforcing previous observations that patient-reported disease activity does not consistently reflect vascular involvement in AS. ^19, 28^ Overall, these results indicate that cross-sectional disease activity measures demonstrated limited and inconsistent relationships with arterial stiffness indices, underscoring the multifactorial nature of vascular alterations in AS.

### Determinants of arterial stiffness in ankylosing spondylitis

Multivariable regression analyses identified body mass index as a consistent independent determinant of arterial stiffness across multiple ultrasound-derived parameters, independent of age, sex, smoking status, and hypertension. This observation underscores the role of metabolic factors in modulating vascular stiffness in AS and is consistent with previous studies reporting associations between arterial stiffness and cardiometabolic characteristics rather than inflammatory indices alone.^28,37^ The predominance of BMI as a predictor over inflammatory markers suggests that non-inflammatory mechanisms, including adiposity-related vascular and metabolic effects, may substantially contribute to arterial stiffening once functional remodelling is established.

### Clinical and methodological implications

From a methodological perspective, the use of one-point echo-tracking technology represents a strength of the present study. Unlike carotid–femoral pulse wave velocity, which integrates heterogeneous arterial segments and relies on external distance measurement, echo-tracking enables precise local assessment of arterial wall biomechanics. Previous comparative studies have shown good agreement between echo-tracking-derived local PWV and conventional cfPWV, supporting the validity of this approach for vascular phenotyping in clinical research.^38^

Clinically, our findings suggest that vascular ultrasound-derived stiffness indices may serve as sensitive markers of early cardiovascular involvement in ankylosing spondylitis, particularly in patients classified as low or intermediate risk by traditional algorithms. Incorporation of arterial stiffness assessment into cardiovascular evaluation protocols for AS could therefore improve risk stratification and facilitate earlier preventive interventions.

### Limitations

Several limitations should be acknowledged. The cross-sectional design precludes causal inference and does not allow assessment of temporal relationships between inflammation, arterial stiffness and structural atherosclerosis. The relatively modest sample size, particularly in the control group, may limit statistical power for detecting small differences in CIMT. A further limitation concerns the lack of assessment of cumulative inflammatory exposure and treatment-related effects on vascular stiffness, factors that may play a substantial role and require confirmation in longitudinal designs. Additionally, formal intra- and interobserver reproducibility analyses were not performed within the present dataset. However, all examinations were conducted by a single experienced operator using a standardised acquisition protocol and integrated semi-automated echo-tracking software with real-time ECG synchronisation and automated border detection. Previous validation studies have demonstrated high reproducibility of one-point carotid stiffness parameters assessed with this methodology. Nevertheless, the absence of internal reproducibility assessment should be considered a limitation and warrants confirmation in future longitudinal studies.

## CONCLUSIONS

In conclusion, patients with ankylosing spondylitis exhibit significant functional vascular impairment characterised by increased arterial stiffness, despite preserved traditional cardiovascular risk profiles and minimal structural atherosclerotic changes. One-point echo-tracking–derived arterial stiffness indices capture early vascular damage beyond conventional risk assessment and may represent valuable tools for improving cardiovascular risk stratification in ankylosing spondylitis. This supports a shift toward integrated vascular imaging approaches for the early detection of inflammation-associated cardiovascular damage in inflammatory rheumatic diseases.
